# Dynamics of internal bore migration within turbidity currents under variable dam slopes

**DOI:** 10.1038/s41598-026-50042-y

**Published:** 2026-04-28

**Authors:** Fong-Zuo Lee, Jihn-Sung Lai, Nafeela Imtiyaz

**Affiliations:** 1 Department of Civil Engineering, National Chung Hsing University, 145 Xingda Rd., South Dist., Taichung City, 402202 Taiwan, R.O.C.; 2 Hydrotech Research Institute, National Taiwan University, Taipei, 10167 Taiwan, R.O.C.; 3 Department of Civil and Environmental Engineering, Monash University, Clayton, VIC Australia

**Keywords:** Muddy lake, Run-up height, Turbidity current, Head velocity, Upstream-migrating internal bore, Engineering, Environmental sciences, Hydrology, Natural hazards

## Abstract

Reservoir sedimentation and turbidity currents present persistent challenges to water resources management by reducing storage capacity, degrading water quality, and increasing risks to hydraulic infrastructure. This study provides a spatiotemporal investigation of submerged muddy lake deformation, focusing on estimating the travel time of turbidity current head velocity between the plunge point and the dam. Such estimates are critical for optimizing sediment venting operations and mitigating sediment-related hazards. Additionally, the run-up height of forward-propagating turbidity currents and the dynamics of upstream-migrating internal bores are examined to evaluate the effects of dam slope and the temporal evolution of muddy lake deformation. A series of laboratory experiments was conducted using three dam slopes, three inflow discharges, and three turbidity current concentrations, during which turbidity current head velocity and internal bore velocity were systematically measured. The results indicate that, for a given concentration and dam slope, the dimensionless bore velocity is largely insensitive to variations in inflow discharge and dam slope. The bore velocity decreases with increasing upstream distance and is consistently approximately 50% lower than the corresponding turbidity current head velocity. Furthermore, the square root of the dimensionless propagation distance is identified as the key governing parameter controlling both the relative bore velocity and the run-up height. Regression analysis demonstrates that the inflow densimetric Froude number, current thickness, and dam slope dominate the flow mechanism of upstream-migrating internal bores, yielding coefficients of determination (R^2^) exceeding 0.70. These findings advance fundamental understanding of muddy lake deformation processes and offer practical insights for managing turbidity currents and reservoir operations. By improving the prediction of turbidity current dynamics and internal bore behavior, this study supports the development of more effective sediment venting strategies. It contributes to the long-term sustainability and safety of reservoir systems.

## Introduction

The annual mean loss of reservoir storage due to sedimentation is already higher than the increase in capacity by constructing new reservoirs for water supply, irrigation, and hydropower^1^. The sustainable use of the reservoir is more important, and long-term storage is not guaranteed^2^. In nature, turbidity currents often transport and deposit fine sediments. Turbidity currents with high sediment concentration mainly happen during floods. It is well recognized that turbidity currents are a type of density current that occurs in lakes, reservoirs, or seas due to concentration differences and that they flow into stagnant water. During heavy rainfall events, the watershed may generate significant sediment yield. The massive sediment-laden flow originates from the upstream river reach and follows the thalweg to the reservoir’s deep zones toward the dam. Three types of turbidity currents can be formed as underflow, interflow, and overflow, depending on variations in sediment concentration relative to ambient water characteristics. If the density difference of the current is due to the presence of suspended load and flow near the bottom (underflow), the current is the case of this study.

**Fig. 1:**
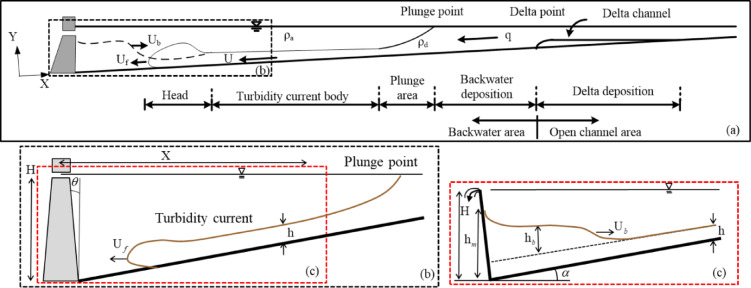
Sketch of (**a**) turbidity current movement, (**b**) run-up height, and internal bore migrating upstream.

As a turbidity current flows into a lake or reservoir, deposition occurs due to a decrease in velocity. In general, the coarser grain size of sediments would deposit quickly and form a delta near the backwater region at the tail of the mainstream reservoir. The delta would finally grow through the flow mechanism, creating a delta stream. The hydraulic phenomenon of this delta region is similar to that in the shallow water of open channels. Sediments would flow through the delta and be sorted for deposition. The intrusion of turbidity currents may dominate sediment deposition, reducing reservoir storage capacity. Given a continuous sediment supply from upstream, the turbidity current approaches the dam. It reflects backward to generate an upstream-migrating internal bore to migrate from the dam to the upstream end of the reservoir. As a result, a submerged, muddy lake can form within a reservoir. In the muddy lake, if sediment cannot be released from the reservoir, it will gradually settle out and consolidate. The downstream head velocity of the turbidity current controls the timing of sediment venting for reservoir siltation operation. However, the velocity of the upstream-migrating internal bore may provide the time required to complete the formation of the muddy lake. The surface of this muddy lake will extend along a nearly horizontal profile upstream from the dam. The volume of the muddy lake will increase, and the interface will rise as long as turbid inflow exceeds losses by venting and the compaction of the solids. Therefore, the formation period of a muddy lake could provide vital information for topographic evolution and sediment management in a reservoir. However, most previous studies on turbidity currents have focused on downstream propagation and head velocity, while upstream-migrating internal bores generated during dam impoundment or consolidation stages remain poorly understood, particularly under variable dam slopes. Figure 1a illustrates the turbidity current moving down the bottom slope angle $\alpha$ with a density of $\rho_{d}$, the unit-width discharge of $q$, the thickness of inflow turbidity current $h$, the average body velocity $U$ of inflow turbidity current, the head velocity toward downstream $U_{f}$, the dam height $H$, the run-up height $h_{m}$, the upstream slope of the dam $\theta$, the velocity of migrating internal bore backward to upstream $U_{b}$, the thickness difference of migrating internal bore $h_{b}$ and under inert water in a reservoir containing water with a density of $\rho_{a}$.

A turbidity current can be described as a sediment–water mixture with a bulk density $\rho_{d}$ that exceeds that of the ambient water by Δρ, producing a gravity-driven downslope flow. The water buoyancy reduces the force of gravity acting on the turbidity current. If the densities of $\rho_{d}$ and $\rho_{a}$ remain constant, the adequate gravitational acceleration ($g^{\prime}$) on the turbidity current can be expressed as:

1 $$g^{\prime} = g\left(\frac{{\rho_{d} - \rho_{a} }}{{\rho_{a} }}\right) = g\frac{\Delta \rho }{{\rho_{a} }}$$

Several field studies and laboratory experiments have been conducted on body thickness, velocity structure, and head velocity toward the downstream of turbidity current in various sections^3–9^. Based on experimental data, Turner^10^ found that the head velocity ($U_{f}$) toward downstream at quasi-uniform width without the effects of bottom friction and mixing process can be expressed as:

2 $$U_{f} = \sqrt {2\left(\frac{{\rho_{d} - \rho_{a} }}{{\rho_{a} }}\right)gh} = \sqrt {2g^{\prime}h}$$

However, the timing of a turbidity current reaching a dam is dominated by the head’s forward velocity. In practice, it is better to estimate the head velocity from the current head height rather than the current body height. Based on the laboratory data, Middleton^11^ found that the head velocity of turbidity current follows Keulegan’s relation^12^ and can be expressed as:

3 $$U_{f} = 0.75\sqrt {g^{\prime } h_{f} }$$

where $h_{f}$ = the height of the turbidity current head.

Altinakar^13^ also proposed a similar formula for small slopes (bed slope < 3%) and presented the coefficient number 0.63 instead of 0.75. Chen et al.^14^ noted that the estimation of head velocity could apply the water depth at the plunge point location instead of the height of the turbidity current body. In addition, based on several experimental tests^4,15,16^, head velocity is defined as a function of inflow turbidity current flux, discharge and bed slope as follows:

4 $$U_{f} /(F_{s} )^{1/3} = f\left(q/\sqrt {g^{\prime}h_{f}^{3} } ,S,\alpha \right)$$

In which, $F_{s} = g^{\prime}_{{}} q$ = turbidity current flux, $S$ = bed slope (fixed to 0.02 in this study).

Turbidity currents are gravity-driven flows generated by density contrasts associated with high sediment concentrations that propagate along the bed. The head velocity of a turbidity current plays a critical role in controlling the distance over which sediment transport and deposition patterns occur. Previous studies have demonstrated that head velocity is primarily governed by gravitational forcing and is further modulated by bed roughness. More recent investigations indicate that particle auto-suspension mechanisms and the distribution of bed shear stress influence the structure of the flow head, thereby regulating its propagation speed and sediment deposition efficiency^17,18^.

When turbidity currents encounter abrupt topographic variations or engineered structures, the flow commonly transitions from supercritical to subcritical conditions. This transition requires rapid adjustments of momentum and flow thickness over a short spatial scale, leading to the formation of internal hydraulic jumps or internal bores^19^. Such processes are fundamental to the development of muddy lakes. As reported by Wang et al.^20^, turbulence intensity within the internal jump region strongly controls sediment mixing efficiency, while local bathymetric variations and sediment resuspension dynamics govern the stability of muddy lakes. Although turbidity currents and internal bores have been extensively studied independently—with emphasis on their generation mechanisms, propagation characteristics, and general hydraulic behavior—their coupled dynamics during interactions with dam structures, as well as the associated run-up processes, remain poorly understood. In particular, quantitative assessments of how inflow conditions and dam slope jointly control momentum transformation, internal bore development, and run-up height are still lacking.

This study clearly states that the main knowledge gap lies in the limited experimental and numerical evidence on the interaction between turbidity currents and internal bores near reservoir dams under controlled hydraulic conditions. The novelty of this study is therefore its integrated approach, combining systematic laboratory experiments with numerical simulations to explicitly characterize the coupling mechanism between turbidity currents and internal bores and to quantify their effects on run-up height under varying dam slopes and inflow discharges.

Many researchers have adopted 2D or 3D numerical models to study the dynamics and impacts of turbidity current movement^21–31^. These simulations generally divide the turbidity current into three distinct parts. The head is the front-most part that is highly turbulent and vulnerable to flow divergence. The second section is a body that is not as turbulent as the head, but in this part of the current, the suspension erosion and deposition happen, and the water’s entrainment. The third section is the tail, which is weakly turbulent. The head is where upward mixing occurs, while the tail is where upward dispersion occurs.

In their study, Hu et al.^32^ used a 2D turbidity current model to evaluate transport rates in a reservoir, concluding that it is a valuable tool for operating sluice gates to release excess sediment. Huang et al.^22^ used a two-dimensional (2D) layer-averaged model to simulate turbidity current characteristics in the Shihmen Reservoir. They identified travel patterns by comparing concentration profiles across transects. Ouillon et al.^26^ conducted highly resolved three-dimensional simulations of turbidity currents on shallow slopes in a stratified saline ambient. They examined changes in front velocity and intrusion depth under varying particle sizes, stratification, and slope angles. Their results indicated that increasing stratification reduces both front velocity and intrusion height. Anari et al.^21^ further supported the 2D model’s suitability for simulating horizontal turbidity current movement, especially near reservoir sluice gates. These studies collectively underline the critical role of sediment transport management in ensuring reservoir storage sustainability.

In addition, some numerical simulations show that obstacles cause local accumulation of density currents, increase flow thickness, and significantly reduce flow velocity. Backflow zones and vortex structures are generated downstream of the obstacles, accelerating energy dissipation and promoting the settling of suspended particles^33^. Experimental studies also indicate that when the flow thickness is less than the obstacle height, the turbidity current cannot directly cross the obstacle and is forced to uplift, accompanied by intense mixing, which substantially weakens its sediment transport capacity^34^. From a management perspective, simulation studies by Xu et al.^35^ and Oshaghi et al.^36^ confirm that the topographic boundary layer strongly constrains the evolution of muddy lakes. By precisely controlling the timing of the sediment-discharge gates, the pressure difference generated by the inrush waves can be used to maximize the sediment-discharge ratio.

Moreover, Hung et al.^37^ applied a 2D turbidity current model to simulate sediment distribution in Shihmen Reservoir and to assess outflow rates and sediment concentrations from desilting facilities. They successfully captured outflow rates and sediment concentrations from desilting structures. Their findings emphasized the precision and practical utility of numerical modelling in understanding turbidity dynamics. Similarly, Huang^38^ and Lee and Huynh Nguyen^25^ demonstrated the model’s validity in predicting turbidity current movement toward dam faces and the outlet sluicing process at different elevations. They also highlighted the importance of mesh resolution and sensitivity to the drag coefficient for accurate simulation results.

However, as mentioned previously, research on the velocity of upstream-migrating internal bores remains limited. Only Lamb et al.^39^ proposed an equation for estimating their velocity in small intraslope basins using shallow-water wave theory. Addressing this gap, the present study investigates the flow characteristics of the run-up height of forward turbidity currents and upstream-migrating internal bores during submerged muddy lake deformation. Experimental results are compared with the more extensively studied head velocity of turbidity currents. A series of laboratory experiments, complemented by a 3D numerical model, was conducted under controlled conditions with varying dam slopes, inflow discharges, and turbidity current concentrations.

By elucidating the interactions between turbidity currents and internal bores, the results facilitate the transfer of knowledge from laboratory-scale experiments to real-world reservoir applications. Therefore, the novelty of this study stems from its integrated approach of combining systematic laboratory experiments with numerical simulations. Specifically, it aims to (1) explicitly characterize the coupling mechanism between turbidity currents and internal bores, (2) quantify their effects on run-up height under varying dam slopes and inflow discharges, and (3) provide validated relationships that extend existing turbidity current research toward practical reservoir and dam engineering applications. These contribute to sediment-related hazard research and support mitigation and prevention for risk-reduction strategies. The insights gained can inform adaptive reservoir operation at the local scale, guide national sediment management planning to enhance flood control and water supply security, and contribute to international discussions on sustainable water resources governance.

## Methods

### Physical model theory

The velocity of a turbidity current is affected by many parameters. The measurable and geometrical parameters are included to develop an equation to estimate the velocity of the upstream-migrating internal bore and the head of the turbidity current in variant slopes of the Dam reaches. In this study, the turbidity current is assumed to be fully developed, and its body thickness and velocity depend on the inflow discharge. This means that measurable parameters, such as inflow discharge, inflow concentration, and other geometric parameters, can be used to describe the velocity of a turbidity current. In addition, because of the mass transfer between the turbidity current and the ambient flow during migration, the Richardson number has been discretized here to analyze an unsteady turbidity current. Therefore, the measurable and geometric parameters majorly contribute to the velocity of the upstream-migrating internal bore $U_{b}$, and the head of turbidity current $U_{f}$, and those parameters can be derived from Eq. (4) and expressed as a function as follows:

5 $$U_{f,b} = f(g^{\prime},q,h_{b} ,S,X,\alpha ,\theta )$$

Here, $X$ denotes the distance measured from the downstream boundary. Based on the dimensional analysis framework and governing parameters identified by Fathi-Moghadam et al.^4^, the resulting dimensionless groups are formulated and expressed in Eq. (6) as follows:

6 $$U_{f,b} /(g^{\prime}q)^{1/3} = f(q/\sqrt {g^{\prime}h_{b}^{3} } ,X,\alpha ,\theta )$$

### Physical model measurement

Experiments were conducted at the Hydrotech Research Institute, National Taiwan University, Taipei, Taiwan. The experimental setup follows that described by Lee et al.^24^, where detailed information can be found. The laboratory measuring system consists of a straight flume with an adjustable bed slope, a width of 0.20 m, and a total length of 20 m. A schematic of the measuring section, together with the dense- and clear-water supply systems, is shown in Fig. 1b. In all experiments, the flume bed slope was fixed at 0.02, and the downstream overflow height was maintained at 0.5 m. Prior to each test, the flume was filled with ambient clear water supplied from a constant-head tank. A separate upstream constant-head tank was used to introduce dense water into the flume, allowing controlled release into the ambient fluid. The dense water was prepared by dissolving salt in clear water and was pumped from a mixing container to the upstream head tank. The volumetric concentration was adjusted to establish the prescribed inflow boundary conditions for each experimental run. To facilitate flow visualization, a red dye was added to the dense water in the mixing container. The turbidity current was then released at the upstream end of the flume and regulated by control valves to ensure steady inflow conditions. Clear water was discharged at the downstream end of the channel to maintain a constant water level. During each experiment, the propagation of the turbidity current head and the velocity of the upstream-migrating internal bore were continuously monitored using a video-based tracking technique. The experiments were formulated after reviewing field reports from Taiwanese dams^40^, selecting typical values, and emphasizing the influence of inflow discharge, concentration, and upstream dam slopes. The velocity of the upstream-migrating internal bore and the head of the turbidity current were measured at three upstream slopes of the Dams ($\theta \,$ = 90, 45, and 21.8 degrees), three different concentrations (volume concentration C = 0.0025, 0.00493, and 0.0075), and three inflow discharges of turbidity current ($q$ = 14.11 m^2^/s, 24.72m^2^/s, and 33.98 m^2^/s). In addition, to highlight head velocity performance, the flume slope is fixed at 0.02^41^. The velocity of the upstream-migrating internal bore and the head of the turbidity current were determined by recording the time taken by the turbidity current head to travel a certain distance in the measuring section. The head velocity, internal bore velocity, and run-up height were determined using video-based tracking techniques. A calibrated scale mounted on the flume wall was used to track the turbidity current’s position, thickness, velocity, and run-up height over time.

### Numerical model simulation

#### Governing equation of fluid

In this study, ANSYS CFX is employed to simulate the behavior of hyperpycnal flow in a flume. Several approaches exist in ANSYS CFX for modelling hyperpycnal flow. The first method involves solving the convection–diffusion equation and the water body equation. This approach estimates sediment distribution and solves the transport equation for the sediment-laden flow. However, this method neglects the momentum exchange between the two phases of the fluid, i.e., the interaction between sediment and ambient water.

The second method involves solving the Lagrangian particle transport equation and the equation of motion for ambient water, enabling a detailed analysis of their spatial distributions. However, this method is computationally intensive and time-consuming.

The third method is the multiphase Volume of Fraction (VOF); and the practical application of this method can be divided into two categories; the first is a multiphase fluid with a straightforward interface, belonging to the continuous-continuous phase, which is suitable for dealing with problems such as free flow surface; the other is a multiphase fluid does not have a straightforward interface between the two phases and belongs to the continuous-disperse phase, where each phase has its own continuous equation and momentum equation, and then the mass exchange and momentum exchange between the phases are considered. The third approach has been adopted in this study.

Turbidity current is a three-dimensional phenomenon. Therefore, a three-dimensional numerical model capable of simulating turbidity currents in the ambient water is required. Hence, a three-dimensional numerical model in ANSYS CFX 2020-R1 has been used. The three-dimensional Reynolds-averaged Navier–Stokes equations and the sediment continuity equation are the governing equations in ANSYS CFX. The standard k-ε model is used in the Navier–Stokes equations to calculate the increase in viscosity due to turbulence and the modification of the settling velocity. The governing equations are as follows^29^:

7 $$\frac{\partial \rho }{\partial t}+\frac{\partial }{\partial {x}_{i}}\left(\rho {U}_{i}\right)=0$$

8 $$\frac{\partial (\rho {U}_{i})}{\partial t}+\frac{\partial \left(\rho {U}_{i}{U}_{j}\right)}{\partial {x}_{j}}=-\frac{\partial p{\prime}}{\partial {x}_{i}}+\frac{\partial }{\partial {x}_{j}}\left[({\mu }_{t}+\mu )\left(\frac{\partial {U}_{i}}{\partial {x}_{j}}+\frac{\partial {U}_{j}}{\partial {x}_{i}}\right)\right]+B{\prime}-{v}_{f}{v}_{f}\frac{\partial }{\partial {x}_{j}}\left(\frac{{\rho }_{w}{\rho }_{s}}{\rho }\right)$$

9 $$\frac{\partial (\rho {c}_{P})}{\partial t}+\frac{\partial (\rho {c}_{P}({U}_{i}))}{\partial t}-\frac{({\mu }_{t}+\mu )}{{\sigma }_{p}}{c}_{P}\left(\frac{\partial {U}_{i}}{\partial {x}_{j}}+\frac{\partial {U}_{j}}{\partial {x}_{i}}\right)+{v}_{f}\frac{\partial }{\partial {x}_{i}}\left({c}_{P}\frac{{\rho }_{w}}{\rho }\right)=0$$

where, *ρ* = mixture density = *(1-c*_*p*_*) ρ*_*w*_ + *c*_*p*_*ρ*_*s*_*; ρ*_*w*_ = water density_*;*_* ρ*_*s*_ = particle density, ${c}_{P}$=local volumetric sediment concentration*;* x_i,j_ = cartesian coordinates ≡ (x, y, z), U_i,j_ = velocity components ≡ (u, v, w), p’ = modified pressure, B’ = [0,0, (* ρ*-ρ_w_) g] is a buoyancy vector*,* g acceleration the turbulence viscosity. The k-ε model assumes that the turbulence viscosity is linked to the turbulence kinetic energy (k) and dissipation (ε) via the relation:

10 $${\mu }_{t}={C}_{\mu }\rho \frac{{k}^{2}}{\varepsilon }$$

where C_μ_ = 0.09 is a constant,

The values of k and ε

11 $$\frac{{\partial \left( {\rho k} \right)}}{\partial t} + \frac{{\partial \rho U_{j} k}}{{\partial x_{j} }} = \frac{\partial }{{\partial x_{j} }}\left[ {\left( {\mu + \frac{{\mu_{t} }}{{\sigma_{k} }}} \right)\frac{\partial k}{{\partial x_{j} }}} \right] + \mu_{t} \left( {\frac{{\partial U_{i} }}{{\partial x_{j} }} + \frac{{\partial U_{J} }}{{\partial x_{i} }}} \right)\frac{{\partial U_{i} }}{{\partial x_{j} }} - \rho \varepsilon$$

12 $$\frac{{\partial \left( {\rho \varepsilon } \right)}}{\partial t} + \frac{{\partial \rho U_{j} \varepsilon }}{{\partial x_{j} }} = \frac{\partial }{{\partial x_{j} }}\left[ {\left( {\mu + \frac{{\mu_{t} }}{{\sigma_{\varepsilon } }}} \right)\frac{\partial \varepsilon }{{\partial x_{j} }}} \right] + \frac{\varepsilon }{k}\left( {\left( {C_{\varepsilon 1} \mu_{t} \left( {\frac{{\partial U_{i} }}{{\partial x_{j} }} + \frac{{\partial U_{j} }}{{\partial x_{i} }}} \right)\frac{{\partial U_{i} }}{{\partial x_{j} }}} \right) - C_{\varepsilon 2} \rho \varepsilon } \right)$$

where C_ε1_ = 1.44, C_ε2_ = 1.92, σ_k_ = 1.0 and σ_ε_ = 1 are constants.

Regarding particle settling behavior, numerous semi-theoretical and empirical relationships for particle fall velocity have been developed and widely applied^42^. In this study, the effective fall velocity of particles in water with suspended sediment concentration is estimated using a hindered settling formulation following Imtiyaz et al.^29^, which is based on the original Richardson and Zaki^43^ relationship and incorporates subsequent modifications proposed by Camenen^44^ and Zhiyao et al.^45^.

#### Numerical model set-up and boundary conditions

The experimental setup is used to create the geometry of the numerical model. The bed slope was set to 2% for the numerical investigation, consistent with the physical model. However, in addition to the three upstream slopes of the dam tested in the physical model, i.e., 90, 45, and 21.8 degrees, two additional slopes, 60° and 75°, were included in the numerical setup. After creating the four flume geometries using Space Claim, the mesh was generated using Ansys Meshing. Since turbidity currents are gravity-driven, they are dense at the bed; the mesh near the flume bed captured this characteristic. The referenced computational meshes and timestep (dt) were constructed similarly to Lee et al.^24^ and Imtiyaz et al.^29^. The representative grid size is less than 0.03 m, and dt is 0.1 s. The total number of mesh elements is 107,006, including 50,090 tetrahedra and 56,916 wedges. The orthogonal mesh quality of our model lies in the range 0.75 to 1, which is considered very good under the orthogonal mesh quality metric spectrum. The Mesh skewness range is 0.0001–0.82, which is also regarded as good.

The simulation was set up using CFX-Pre. We set the material type in this; although CFX-Pre-includes a library of materials, it does not include saline, the fluid used in the flume experiment. Therefore, we create a new material, Saline, with a density of 1023.6 kg/m^3^. Under the material, we specify two properties: density, which comes under the equation of state, and dynamic viscosity, which comes under transport properties. Since the saline solution’s density is close to that of water, we set its dynamic viscosity equal to that of water. However, if a constant value is not available, the CEL editor can be used to define an algebraic expression model instead. Since we have considered a buoyant model, we see the buoyant vector in the Eq. (8), indicating that the effect of gravity is activated using a buoyancy option. We have set the gravitational acceleration in the z direction (based on our model’s coordinate system). Since no heat transfer is modeled in our simulations, a constant temperature of 20 °C is specified, making the model isothermal.

The specified boundary conditions significantly affect the solver’s performance; therefore, careful selection is essential. For model initialization, the initial condition is a Cartesian velocity field with all components set to 0 m/s, a static pressure of 0 Pa, and a sand mass fraction of 0. The boundary conditions can be specified at the inlet and outlet for velocity, mass flow rate, or pressure. For the inlet, the velocity and concentration distributions are specified as boundary conditions. In contrast, the outlet is treated as an opening, and a static pressure condition is imposed on it. A no-slip wall boundary condition is imposed on the side walls and the bottom of the flume, implying that the wall influences the flow and induces velocity gradients near the wall. In contrast, the top wall is assigned a free-slip condition, implying it does not affect the flow.

## Results and discussion

### Numerical model verification

**Fig. 2:**
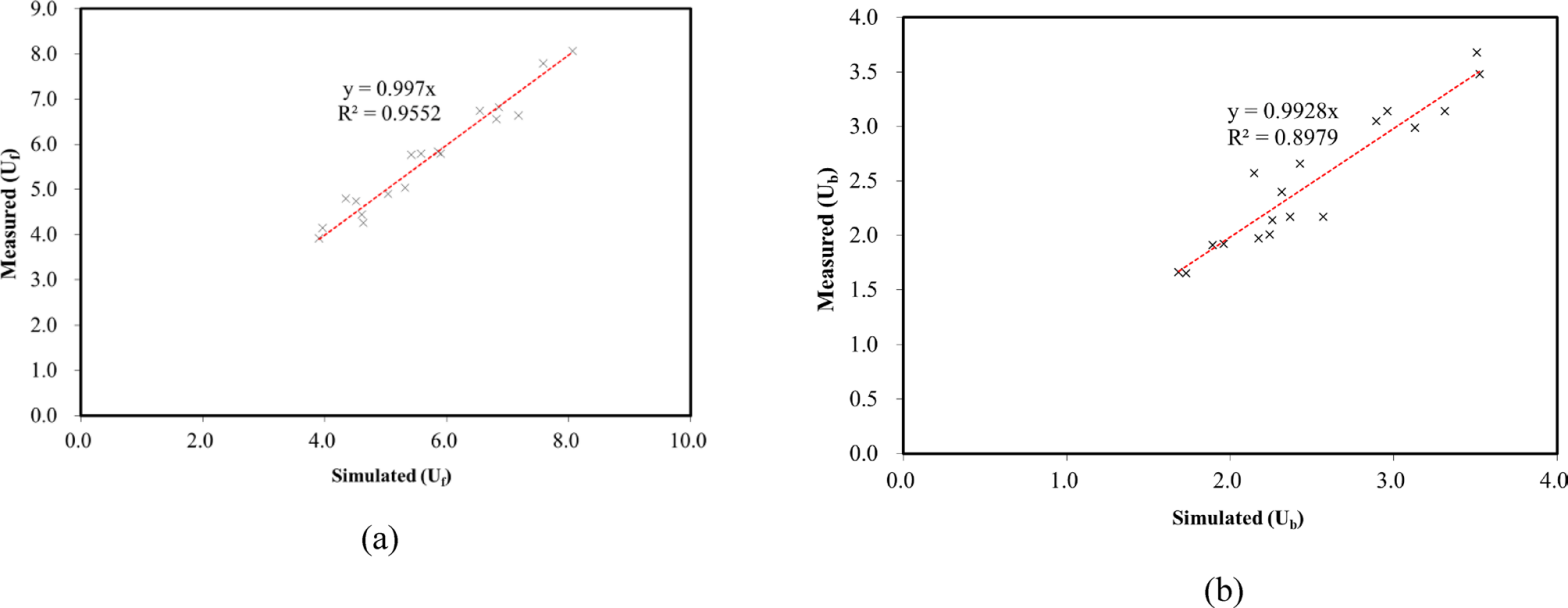
Numerical model verification of (**a**) the head velocity of the turbidity current, (**b**) the upstream-migrating internal bore velocity.

The head velocity of the turbidity current and the velocity of the upstream-migrating internal bore were measured by recording the time taken by the head to traverse a specific section in the flume. Different scenarios were tested for both the turbidity current head velocity and the upstream-migrating internal bore velocity at dam angles of 45° and 90°, and the results were compared using regression analysis, as shown in Fig. 2. Although a comprehensive uncertainty analysis of the experimental and numerical results is beyond the scope of the present study, quantitative performance metrics are provided to assess model reliability. The coefficients of determination (R^2^) are 0.96 for the turbidity current head velocity and 0.90 for the upstream-migrating internal bore velocity. In addition, the average RMSE, MAE, and relative error are 0.23, 0.23, and 0.01, respectively. These comparisons demonstrate good agreement between the experimental observations and numerical simulations, supporting the use of the model to analyze the relationship between upstream-migrating internal bore velocity and inflow conditions.

### Relationship between the velocity of the upstream-migrating internal bore and the inflowing condition

In the laboratory, for a particular flume with a known slope and degree of expansion, the discharge of the turbidity current has been shown to significantly affect its head velocity^4^. Based on observations from large dam reservoirs in Taiwan, turbidity currents with high discharge can reach the dam site, whereas those with low discharge lack sufficient momentum. After the head of the turbidity current arrives at the dam site, the velocity of the upstream-migrating internal bore depends on energy loss and the turbidity current’s inertial forces. Upon arrival at the dam site, the turbidity current ascends the dam slope. When gravity, resistance, and inertial force reach equilibrium, the turbidity current ceases its ascent and begins to descend. This process generates a positive surge that propagates upstream, ultimately causing turbidity within the reservoir. In this study, the velocity of the upstream-migrating internal bore is investigated on a fixed bottom slope (S = 0.02) and varied upstream dam slopes, with three inflow concentrations and three inflow discharges.

**Fig. 3:**
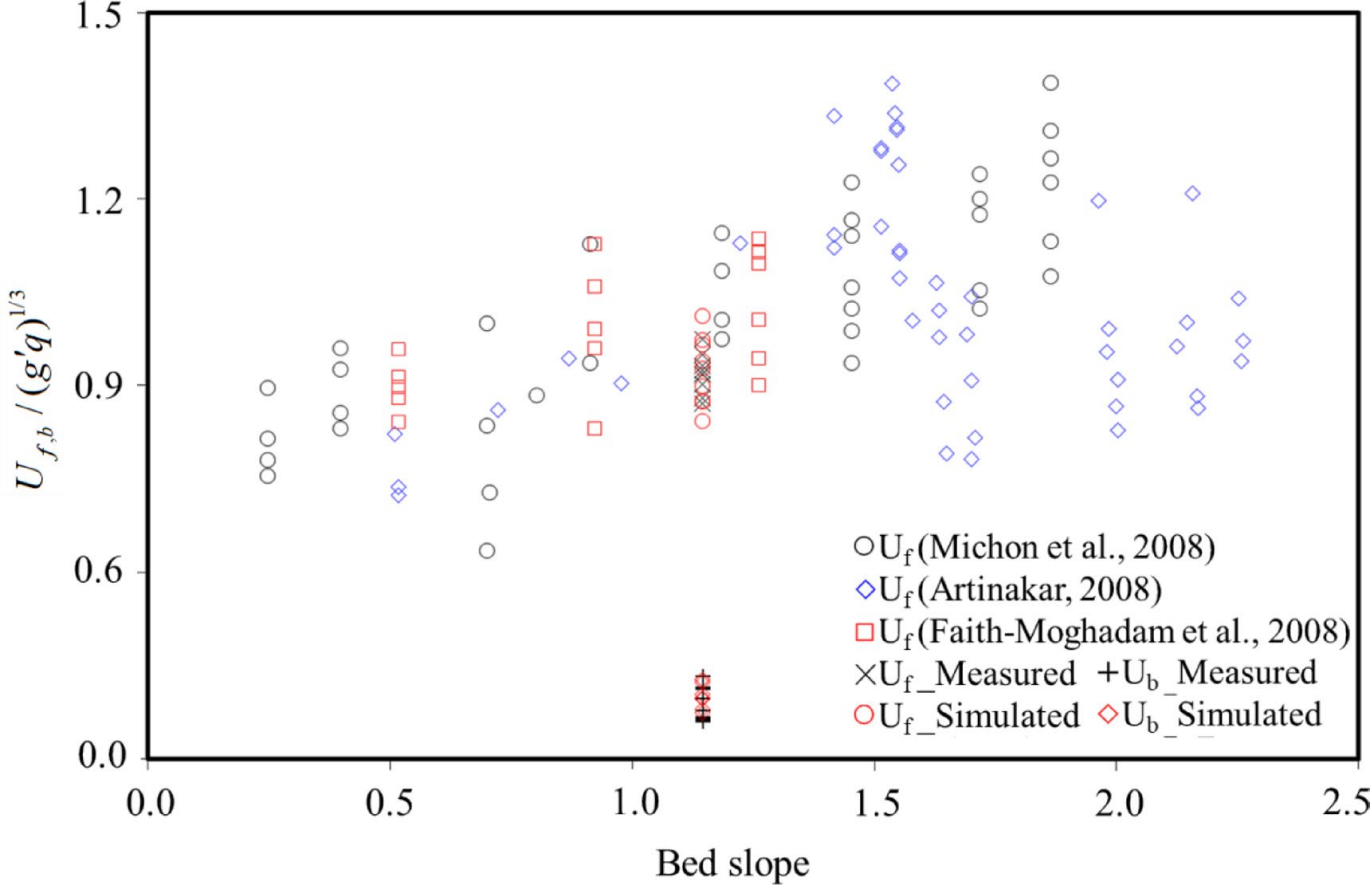
Velocity variations of different flume slopes for a constant cross-section from previous studies.

**Fig. 4:**
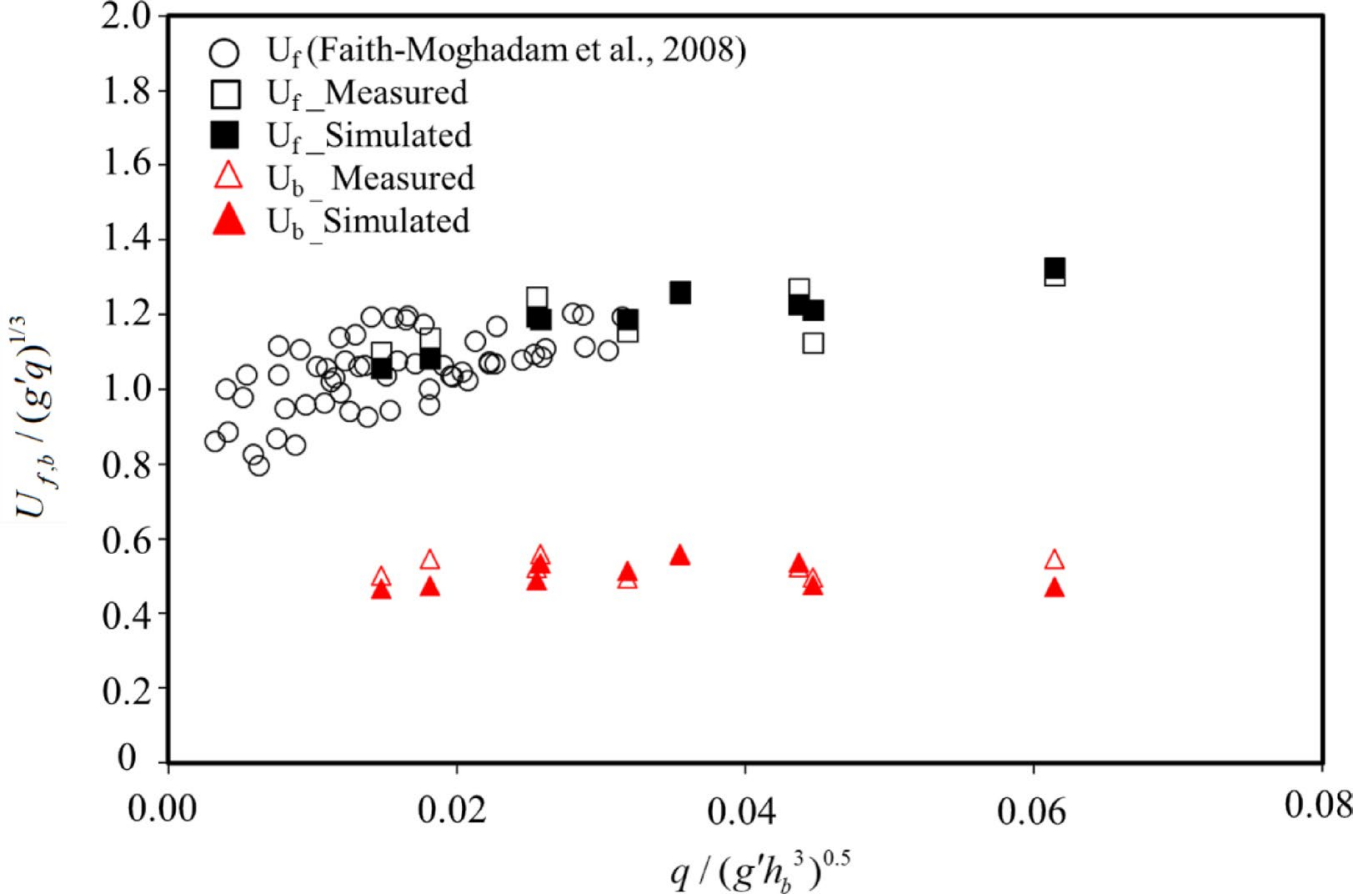
Dimensionless velocity of the head and upstream-migrating internal bore of turbidity current with different inflow discharges.

According to Eq. (6), the most important parameters affecting the velocity of the head and upstream-migrating internal bore of a turbidity current under varying upstream dam slopes are inflow discharge, inflow concentrations, and propagation distance from the downstream side of a turbidity current. Experimental results from previous studies on the variation of the downstream head velocity with slope are illustrated in Eq. (4). The results of the uniform and constant cross-section tests in this study (width 0.2 m) for three upstream slopes of the Dams ($\theta \,$ = 90°, 45°, and 21.8°) are superimposed in Fig. 3, and the head velocity of the turbidity current agrees with that reported in previous studies. Figure 4 presents the dimensionless velocity of the turbidity current head and upstream-migrating internal bore under different inflow discharges.

**Fig. 5:**
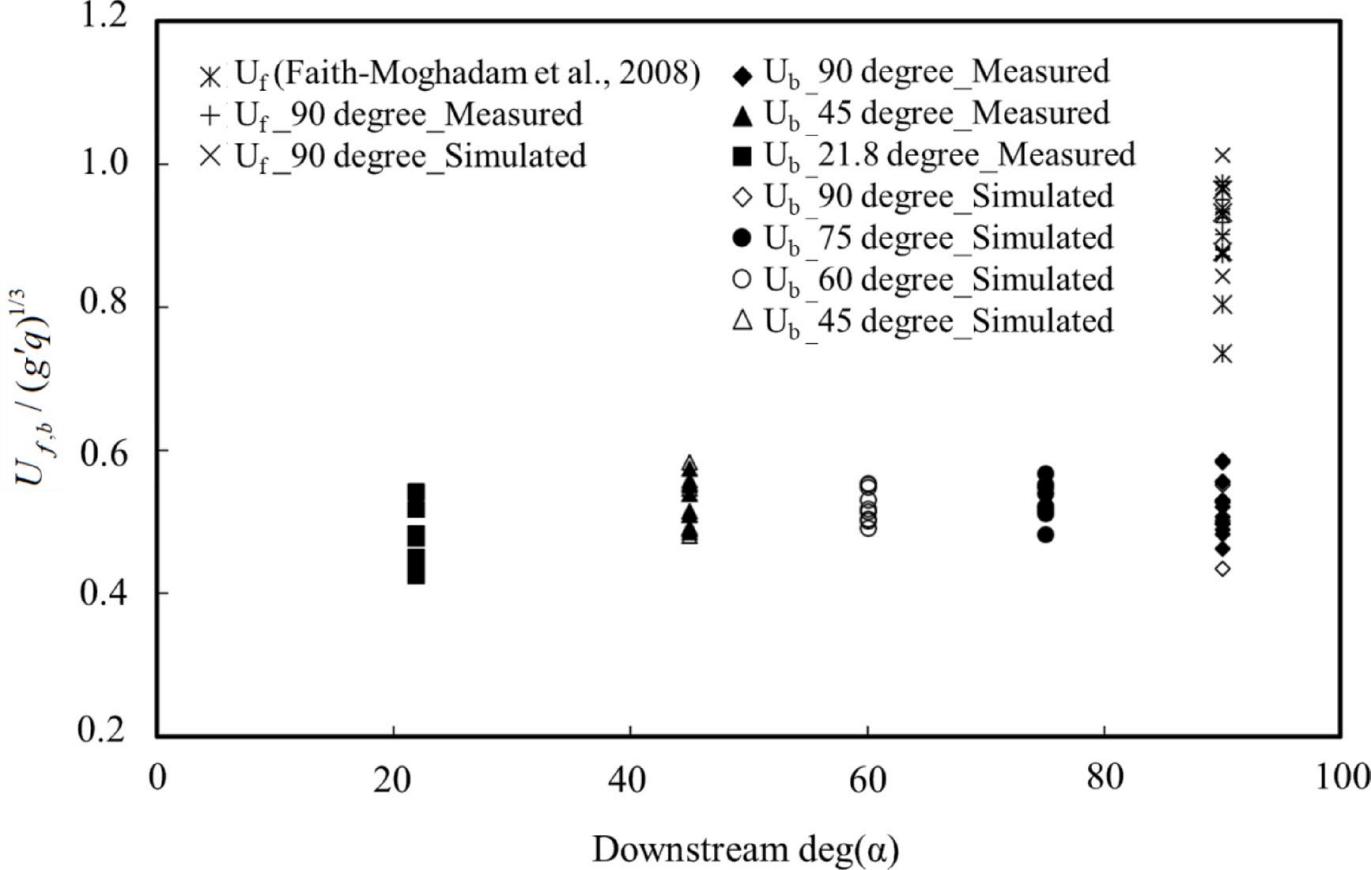
Dimensionless velocity of the head and upstream-migrating internal bore of turbidity current with variant upstream slopes of the Dams.

Compared with the head velocity of the turbidity current, this study does not show a significant correlation between the velocity of the upstream-migrating internal bore and increasing inflow discharge. This means that the velocity of the upstream-migrating internal bore does not increase significantly with inflow discharge. One of this study’s main objectives is to propose a relationship between the velocity of the upstream-migrating internal bore and the inflow boundary conditions, and the variant upstream slopes of the dam, where the dams are constructed in different shapes. Figure 5 illustrates the velocity variation of the turbidity current head and the upstream-migrating internal bore under different upstream dam slopes, at a constant bed slope of 0.02, for various inflow discharges. It shows that the head velocity of the turbidity current is similar to the previous studies, and the velocity of the upstream-migrating internal bore is expectedly lower. The velocity values of the upstream-migrating internal bore are proportional to half of the head velocity of the turbidity current. Figure 5 also indicates that the dimensionless velocity of the upstream-migrating internal bore remains relatively unchanged with varying inflow discharges and upstream dam slopes. Based on this point, the dimensionless velocity of the upstream-migrating internal bore either changes by inflow concentration or propagation distance^46^. Therefore, the correlations and relationships of the head velocity of turbidity current and turbidity current discharge for three pairs of inflow concentrations are tabulated in Table 1. Table 1 lists individual values of the boundary conditions, the head velocity, and the upstream-migrating internal bore of a turbidity current with variant body thicknesses.

**Table 1:** Parameters related to the dynamics of the head velocity for all turbidity current runs.

Inflow $q$ (cm^2^/s)	Inflow volume concentration C	Inflow g’ (m/s^2^)	Inflow h (cm)	DownstreamH (cm)	$U_{f}$(cm/s)					$U_{b}$(cm/s)				
					(90 deg.) (Measured/ Simulated)	(75 deg.) (Simulated)	(60 deg.) (Simulated)	(45 deg.) (Measured/ Simulated)	(21.8 deg.) (Measured)	(90 deg.) (Measured/ Simulated)	(75 deg.) (Simulated)	(60 deg.) (Simulated)	(45 deg.) (Measured/ Simulated)	(21.8 deg.) (Measured)
14.11	0.00250	0.053	5.81	50	4.14/3.97	4.24	4.15	3.92/3.91	3.64	1.65/1.73	1.71	1.68	1.66/1.68	1.47
14.11	0.00493	0.105	5.77	50	4.26/4.63	4.72	4.63	4.44/4.61	4.58	1.97/2.17	2.27	2.25	2.01/2.24	1.81
14.11	0.00750	0.159	5.25	50	4.90/5.04	5.14	5.18	5.04/5.32	4.96	2.17/2.36	2.43	2.32	2.40/2.32	2.01
24.72	0.00250	0.053	9.14	50	4.74/4.51	4.37	4.46	4.80/4.35	4.77	1.92/1.96	1.90	2.04	1.91/1.89	1.77
24.72	0.00493	0.105	7.00	50	5.84/5.86	5.74	5.73	5.77/5.42	5.94	2.572.15	2.53	2.62	2.66/2.43	2.39
24.72	0.00750	0.159	5.75	50	6.56/6.82	6.84	6.73	6.74/6.55	6.90	2.99/3.13	3.07	3.15	3.14/3.31	2.93
33.98	0.00250	0.053	12.26	50	5.79/5.91	5.57	5.74	5.79/5.58	5.79	2.17/2.57	2.49	2.21	2.14/2.26	1.86
33.98	0.00493	0.105	8.00	50	6.64/7.18	6.91	6.91	6.82/6.86	6.74	3.05/2.89	2.86	2.76	3.15/2.96	2.61
33.98	0.00750	0.159	6.76	50	8.06/8.06	8.14	7.89	7.79/7.59	8.00	3.68/3.51	3.47	3.49	3.48/3.53	3.42

**Fig. 6:**
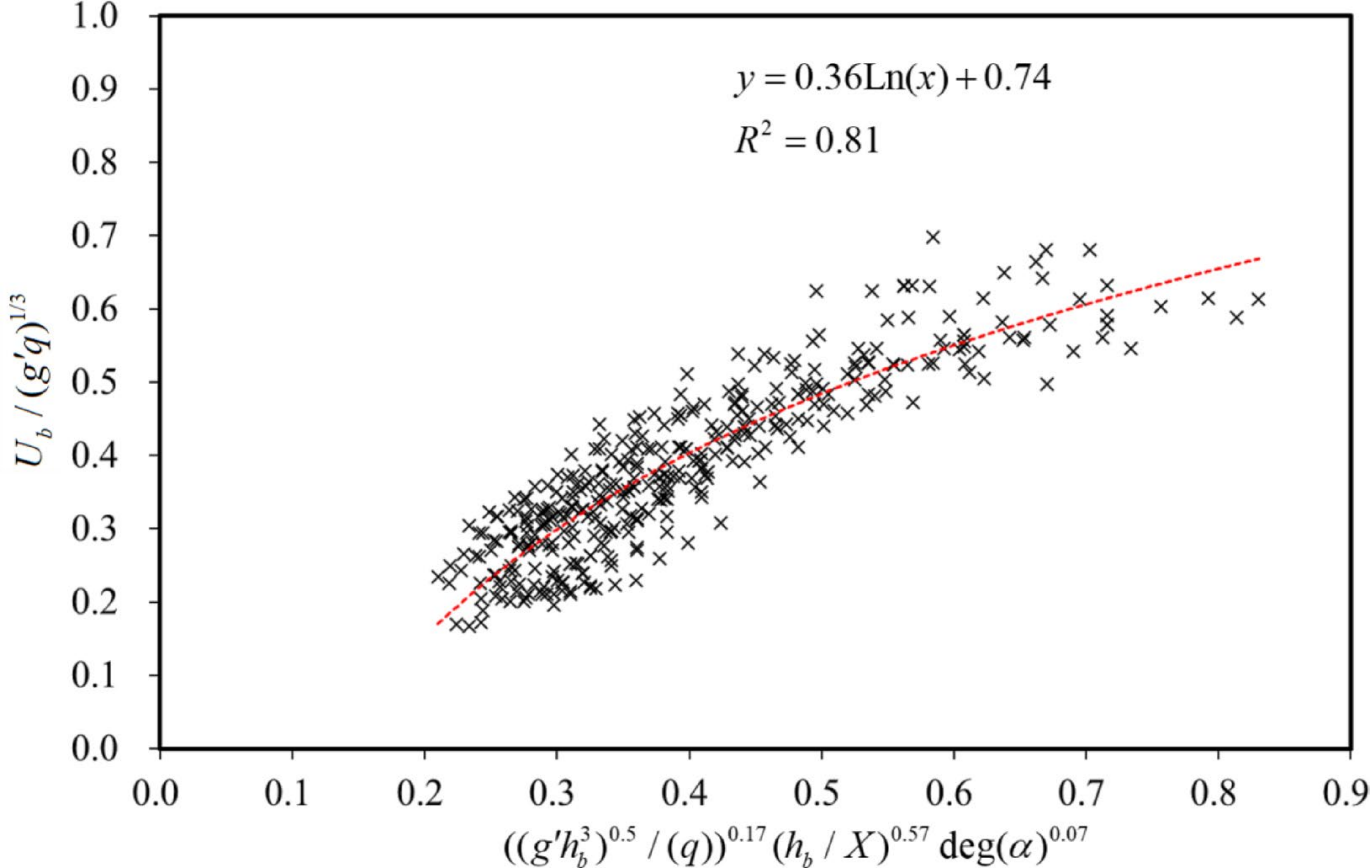
Relationship between upstream-migrating internal bore and propagation distance for variant upstream slopes of the Dams of flume.

However, experimental results show that the velocity of the upstream-migrating internal bore decreases with increasing propagation distance, as shown in Fig. 6. The dimensionless description of the upstream-migrating internal bore is expressed with discharge, propagation distance (the *h*_*b*_ is adapted for the dimensionless distance *X*), and radius of dam slopes in the Eq. (13).

13 $$U_{b} /{(}g{\prime} q)^{1/3} = 0.{36} \times {\mathrm{L}}n\left\{ {\left. {[(g^{\prime } h_{b}^{3} )^{0.5} /q]^{0.17} (h_{b} /X)^{{0.5{7}}} \deg (\theta )^{0.07} } \right\}} \right.{ + 0}{\mathrm{.74,}}\;{\mathrm{R}}^{2} = 0.81$$

Owing to energy dissipation during propagation, the velocity of the upstream-migrating internal bore decreases by approximately 50%, as shown in Figs. 3 and 4. In contrast, the head velocity of the turbidity current is governed primarily by the imposed flume slope of 0.02. This observed decay in bore velocity differs from the findings of Lamb (2004), who reported that bore propagation over a horizontal bed tends to maintain an approximately constant shallow-water wave speed. The discrepancy can be attributed to the present experimental conditions, which include an inflow discharge *q* ranging from 14.11 to 33.98 cm^2^/s, an inflow volume concentration C between 0.0025 and 0.0075, and a turbidity current thickness h between 5.25 and 12.26 cm.

### Velocity relationship between the upstream-migrating internal bore and the head of the turbidity current

Based on the propagation distance and the incoming discharge of the turbidity current, a power relationship between the upstream-migrating internal bore and the inflowing condition is established in Eq. (13), with a constant coefficient. In addition, the approximated velocity magnitude of the upstream-migrating internal bore is proportional 50% to the head velocity of the turbidity current, as shown in Figs. 3 and 4. Therefore, the velocity relationship between the upstream-migrating internal bore and the head of the turbidity current is analyzed in relation to propagation distance in Eq. (14). A power-law relationship is suggested to describe dimensionless velocity changes of the turbidity current at individual locations.

14 $$U_{b} /U_{f} = \varphi \times Ln\left( {\frac{{h_{b} }}{X}} \right)^{{}} + \psi$$

where $\varphi$ = coefficient; $\psi$ = constant.

**Fig. 7:**
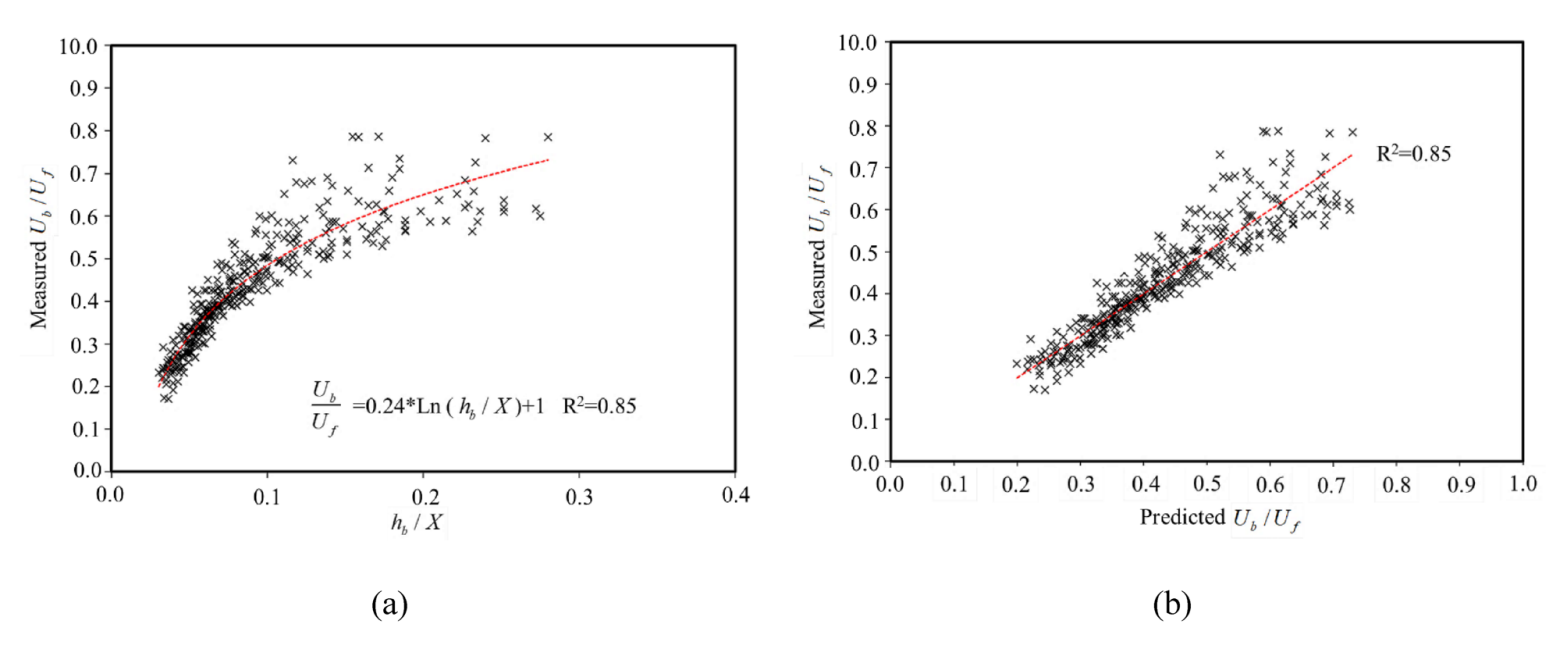
Relationship between upstream-migrating internal bore and the head of turbidity current (**a**) based on h_b_/X (**b**) comparison between predicted and measured data.

Figure 7a shows the regression results between $U_{b} /U_{f}$ and $\frac{{h_{b} }}{X}$ with the number of coefficients “$\varphi$” and the constant “$\psi$”, respectively. Based on the regression investigation, the magnitude of the power index “$\psi$” is approximated to 1.0. Therefore, the modified Eq. (15) is presented, and the number of coefficients “$\varphi$” is regressed to 0.24.

15 $$U_{b} /U_{f} = {0}{\mathrm{.24}} \times Ln\left( {\frac{{h_{b} }}{X}} \right)^{{}} { + 1}$$

Figure 7b compares predicted values using Eq. (15) and measured values of experiments. It shows good agreement results and confidence in the R^2^ value.

### Run-up height of forward turbidity current

After the turbidity current impacts the dam, it continues to ascend along the dam slope until the forces of gravity, friction, and inertia reach equilibrium. At this point, the turbidity current achieves its maximum run-up height. When analyzing the dam slope at 0°, the turbidity current experiences no climbing behavior due to the absence of obstacles. However, with a vertical dam slope ($\theta \,$ = 90°), a vertical barrier forms, causing the turbidity current to climb along the dam. Thus, it can be predicted that the runup height of the turbidity current increases with steeper dam slopes. Experimental observations indicate that, under identical inflow conditions, when the turbidity current does not overtop the tailwater board of the dam, a steeper dam slope promotes a greater conversion of horizontal inertial momentum into a vertical component, resulting in a higher run-up height. The run-up height is therefore controlled not only by the dam slope but also by the inflow discharge, as evidenced by the results summarized in Table 2. These findings are valid within the tested range of slopes and under the specific experimental conditions considered, within which the run-up height generally increases with the dam slope.

**Table 2:** Parameters related to the run-up height of forward turbidity current.

Inflow $q$ (cm^2^/s)	Inflow volume concentration C	Inflow g’ (m/s^2^)	Inflow h (cm)	DownstreamH (cm)	h_m_/H						
					Measured	Simulated	Simulated	Simulated	Measured	Simulated	Measured
					(90 deg.)		(75 deg.)	(60 deg.)	(45 deg.)		(21.8 deg.)
14.11	0.00250	0.053	5.81	50	0.76	0.76	0.74	0.69	0.65	0.65	0.63
14.11	0.00493	0.105	5.77	50	0.67	0.67	0.65	0.64	0.61	0.61	0.58
14.11	0.00750	0.159	5.25	50	0.61	0.61	0.59	0.58	0.56	0.56	0.53
24.72	0.00250	0.053	9.14	50	*	*	0.99	0.98	0.98	0.97	0.94
24.72	0.00493	0.105	7.00	50	0.88	0.89	0.87	0.86	0.85	0.85	0.82
24.72	0.00750	0.159	5.75	50	0.78	0.78	0.75	0.73	0.71	0.71	0.68
33.98	0.00250	0.053	12.26	50	*	*	*	*	*	*	*
33.98	0.00493	0.105	8.00	50	*	*	0.97	0.95	0.93	0.92	0.84
33.98	0.00750	0.159	6.76	50	0.96	0.96	0.89	0.88	0.87	0.84	0.83

*The maximum run-up height hm is higher than the H.

In this study, the run-up height is defined as the height to which the turbidity current rises due to inertia after impacting the downstream dam. Once equilibrium among gravity, friction, and inertia is achieved, the subsequent increase in turbidity current body thickness is influenced by upstream inflow conditions. Experimental and simulated results (as shown in Table 2) demonstrate that under identical inflow rates and concentrations, the maximum run-up height of the turbidity current is proportional to the dam slope (θ). Furthermore, for the same dam slope and unit-width inflow rate, a higher inflow concentration leads to a greater gravitational pull per unit volume, resulting in a reduced run-up height. This indicates that the maximum run-up height is inversely proportional to the inflow concentration, emphasizing gravity as the primary influencing factor regardless of the unit-width inflow rate.

**Fig. 8:**
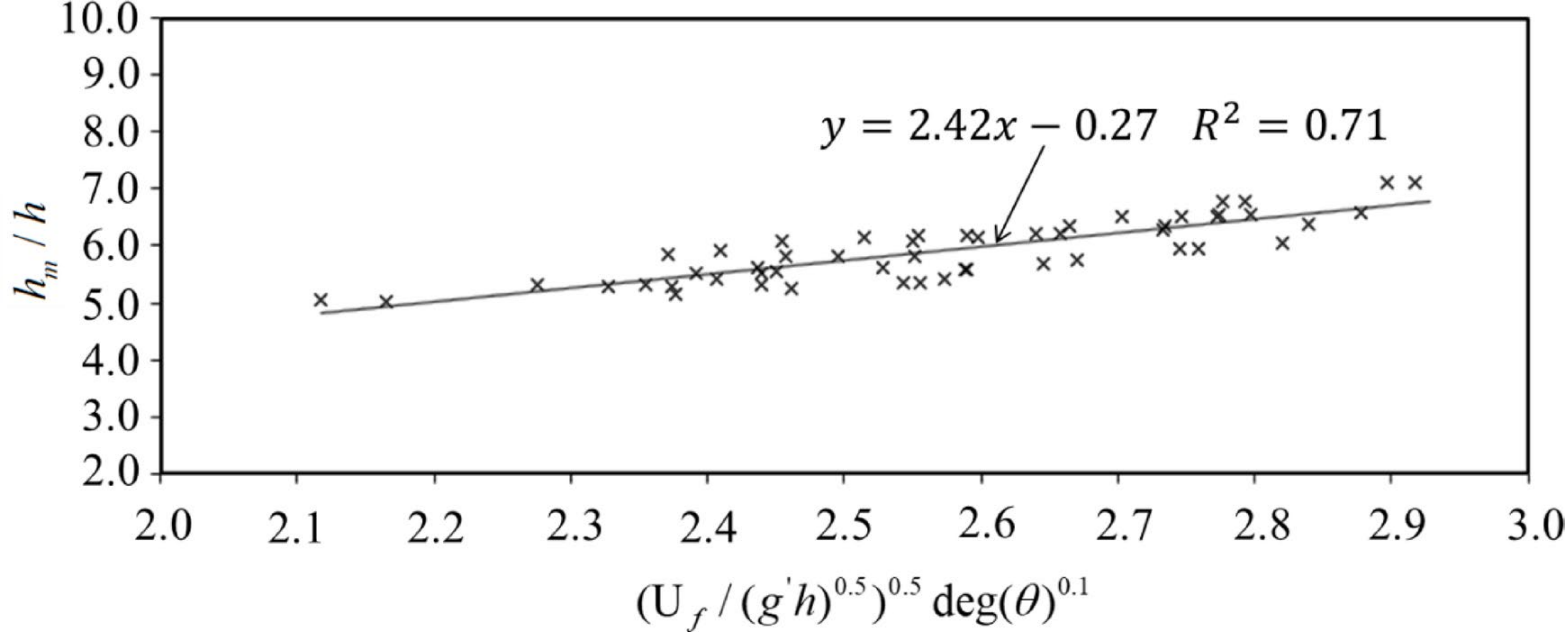
Relationship between forward turbidity current and run-up height.

Additionally, when turbidity currents form in reservoirs and are not immediately vented at the dam, and the turbid water inflow persists, the reservoir evolves into a muddy lake, causing the water level to rise continuously. To understand the change in headwater height over time after the formation of a muddy lake at the dam front, experiments were conducted with θ = 45° under three different inflow concentrations and three different unit-width inflow rates. The results, shown in Fig. 8, reveal that the headwater height of the turbidity current gradually increases over time. A relationship is proposed between inflow turbidity, current body thickness, and run-up height, as shown in Eq. (16).

16 $$h_{b} /h = {2}{\mathrm{.42}} \times (U_{f} /{(}g^{\prime } h{)}^{{{0}{\mathrm{.5}}}} {)}^{{{0}{\mathrm{.5}}}} \times \deg {(}\theta {)}^{0.1} - 0.27,\;{\mathrm{R}}^{2} = 0.71$$

## Conclusions

This study integrated physical and numerical modeling to characterize turbidity current movement along a fixed slope, establishing quantitative relationships for the velocities of both the current head and the upstream-migrating internal bore. The flow characteristics of these bores during the submerged muddy lake deformation period were investigated and compared with the head velocity. Experimental flume setups incorporated parameters such as inflow concentrations and dam slopes, the latter of which reflect typical concrete or earth-rock-fill dam geometries in Taiwan. Furthermore, a 3D numerical model was employed to simulate various inflow conditions and slopes, complementing the experimental data. The integrated results provide a robust basis for analyzing the behavior of internal bores migrating upstream within turbidity currents.

Based on the experimental data summarized in Tables 1 and 2, the velocities of the turbidity current head and the upstream-migrating internal bore are determined for specific inflow concentrations and upstream dam slopes. The experimentally derived head velocities can be validated against field measurements to improve the interpretation of reservoir responses to turbidity inflows, thereby supporting more informed water resources management decisions. Understanding the propagation and distribution of turbidity currents is critical for optimizing reservoir operations, mitigating sedimentation, and safeguarding water quality.

While previous studies on upstream-migrating internal bores remain limited—most notably Lamb et al.^39^, who proposed an equation for small intraslope basins using shallow-water wave theory—this study advances knowledge by examining their dynamics during the submerged muddy lake deformation period and comparing them with the more extensively studied head velocity of turbidity currents. Results show that flow characteristics vary under different run-up heights of forward turbidity currents and upstream-migrating internal bores. The bore’s velocity decreased with increasing upstream distance and was consistently about 50% lower than the turbidity current head velocity. The square root of the dimensionless propagation distance emerged as the dominant parameter controlling the bore’s relative velocity, characterized by a logarithmic relationship. The height difference between the bore and turbidity current body was also critical: higher inflow rates elevated headwater heights, whereas increased concentrations reduced them due to enhanced mixing and diffusion.

However, Eqs. (15) and (16) are derived solely from the dataset obtained in this study and has not been externally validated. We therefore acknowledge that it represents a site- and condition-specific empirical relationship. Accordingly, Eqs. (15) and (16) are presented as a preliminary predictive tool intended to capture relative trends, rather than as a universally applicable formulation. More broadly, clearly identifying the study’s limitations is essential for the appropriate interpretation and application of the results. The revised manuscript explicitly addresses these limitations, which include: (1) scale effects inherent to laboratory experiments, such as the difficulty of simultaneously satisfying Reynolds number and densimetric Froude number similarity, potential distortion of turbulence structures, and modified sediment settling behavior at reduced scales; (2) limitations in sediment representation, as the use of uniform or simplified sediment properties cannot fully reflect the natural heterogeneity, gradation, and cohesion of reservoir sediments; and (3) constraints on the transferability of the findings to prototype reservoirs, noting that the results may not be directly applicable to systems with complex geometries, highly variable inflow conditions, or different density current regimes. Finally, we emphasize that the experimental and numerical results are intended to provide mechanistic insight and identify relative trends rather than to serve as direct quantitative predictions at the field scale. Site-specific calibration and validation are therefore required before the proposed relationships can be applied in practical engineering contexts.

Despite these constraints, the findings not only deepen the understanding of muddy lake deformation processes but also contribute to sediment-related hazard research. By providing scientific insights into reservoir management, this study facilitates knowledge transfer and supports the development of more effective sediment-venting strategies for sustainable operations.

## Data Availability

Data is provided within the manuscript.
